# Supplementary material for: Lahaul–Zanskar–Sham Valley Corridor in Indian Trans Himalayan Region Facilitates Dispersal and Gene Flow in Himalayan Ibex
Source: Biology (Basel). 2023 Feb 28;12(3):382. doi: 10.3390/biology12030382 (PMC10045165; doi:10.3390/biology12030382)
Supplement: Supplementary file 1 [file biology-12-00382-s001.zip › biology-2175570-supplementary.pdf]

Supplementary Information of Lahaul–Zanskar–Sham Valley Corridor in Indian Trans Himalayan Region Facilitates Dispersal and Gene Flow in Himalayan Ibex

Gul Jabin [1,2], Stanzin Dolker [1,2], Bheem Dutt Joshi [1], Sujeet Kumar Singh [1,†], Kailash Chandra [1], Lalit Kumar Sharma [1] and Mukesh Thakur [1,*]

[1] Zoological Survey of India, New Alipore, Kolkata 700053, West Bengal, India
[2] University of Calcutta, Kolkata 700019, West Bengal, India
* Correspondence: thamukesh@gmail.com
† Current address: Amity University, Sector 125, Noida 201301, Uttar Pradesh, India

Table of Contents:

Figure S1: A) Jack knife test, using AUC on test data. B) estimates of relative contributions of the environmental variables to the MaxEnt model. 2
Figure S2 Haplotypes plotted on the spatial map displaying the sharing of haplotype across the landscape 3
Figure S3 Delta K and mean probability of Himalayan Ibex population in the STRUCTURE. 4
Figure S4 DAPC Plot showing LA and LS distribution 4
Figure S5 A) Migration rate between the Lahaul-Spiti and Ladakh region by BAYESASS with 0.05 prior of migration rate. B) Mantel test shows Isolation by distance and display not significant relationship between the geographical and genetic distance. 5

Table S1: Characteristics and reference of microsatellite markers used in standardisation of genotypes in Ibex.**Error! Bookmark not defined.**
Table S2: Sixteen microsatellite marker panels used for multilocus genotyping of Ibex samples 7
Table S3: Total variables used in Spatial modelling of Habitat suitability of Himalayan Ibex 7
Table S4: Polymorphic sites of each haplotype using the d-loop gene 9
Table S5: Haplotype diversity indices and Neutrality test of Ibex using Cytb and d-loop gene. 10
Table S6: Population genetics indices using ten microsatellite marker in two inferred populations LA-Ladakh and LS-Lahaul-Spiti 10
Table S7: First generation migrant individuals which were inferred in different cluster and assigned to another cluster based on Log(L_home/L_max) probability in GeneClass .

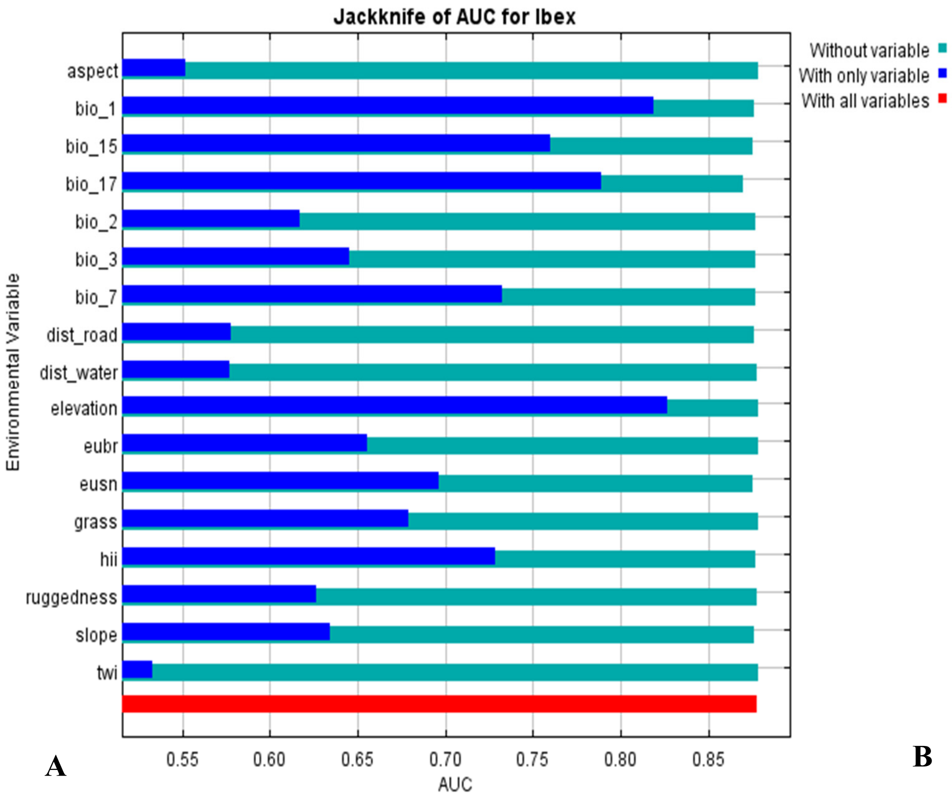

A

B

Variable	Percent contribution	Permutation importance
grass	29.3	0.9
bio_17	20.9	32.1
elevation	12.3	9.6
bio_15	10.4	21.4
bio_2	6.3	3.1
bio_3	4.4	3.2
bio_7	3.6	1.7
bio_1	2.9	4.7
eubr	1.9	1
dist_road	1.7	3.8
slope	1.1	2.6
hii	1.1	2.3
ruggedness	1	1.4
aspect	0.9	2.2
eusn	0.8	7.5
dist_water	0.7	1.1
twi	0.6	1.4

Figure S1: A) Jack knife test, using AUC on test data. B) estimates of relative contributions of the environmental variables to the MaxEnt model.

The environmental variable with highest gain when used in isolation is bio_17, which therefore appears to have the most useful information by itself. The environmental variable that decreases the gain the most when it is omitted is bio_17, which therefore appears to have the most information that isn't present in the other variables.

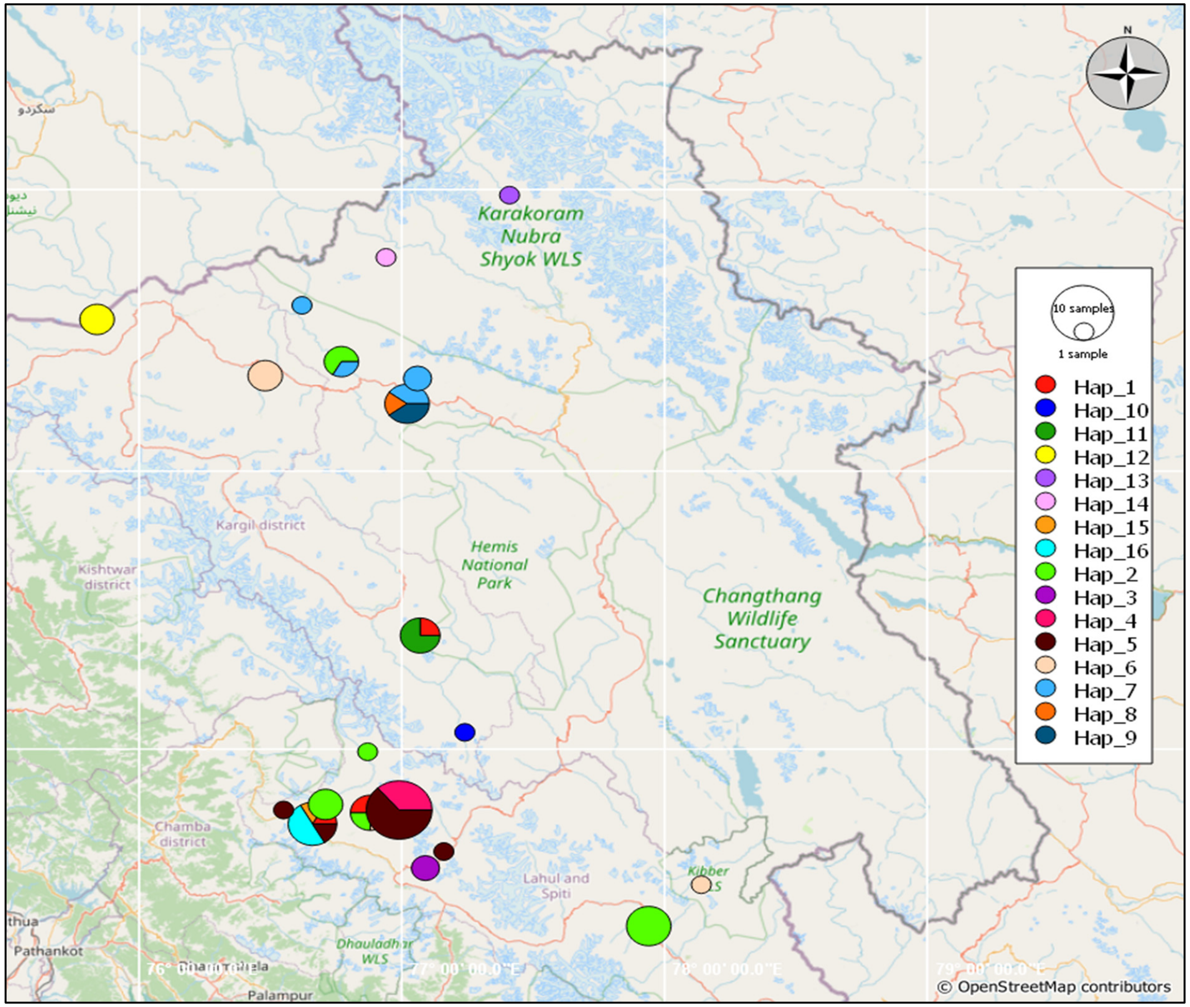

Figure S2 Haplotypes plotted on the spatial map displaying the sharing of haplotype across the landscape

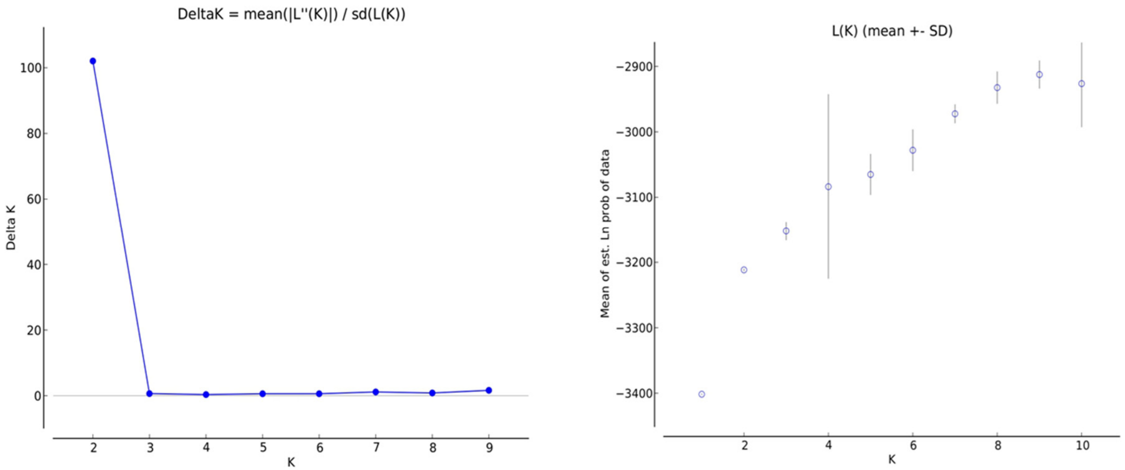

Figure S3 Delta K and mean probability of Himalayan Ibex population in the STRUCTURE.

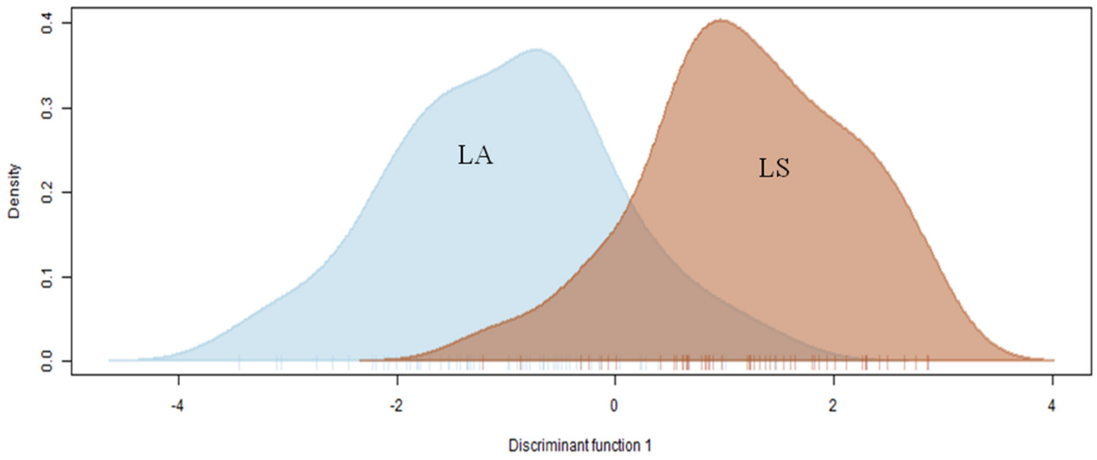

Figure S4 DAPC Plot showing LA and LS distribution

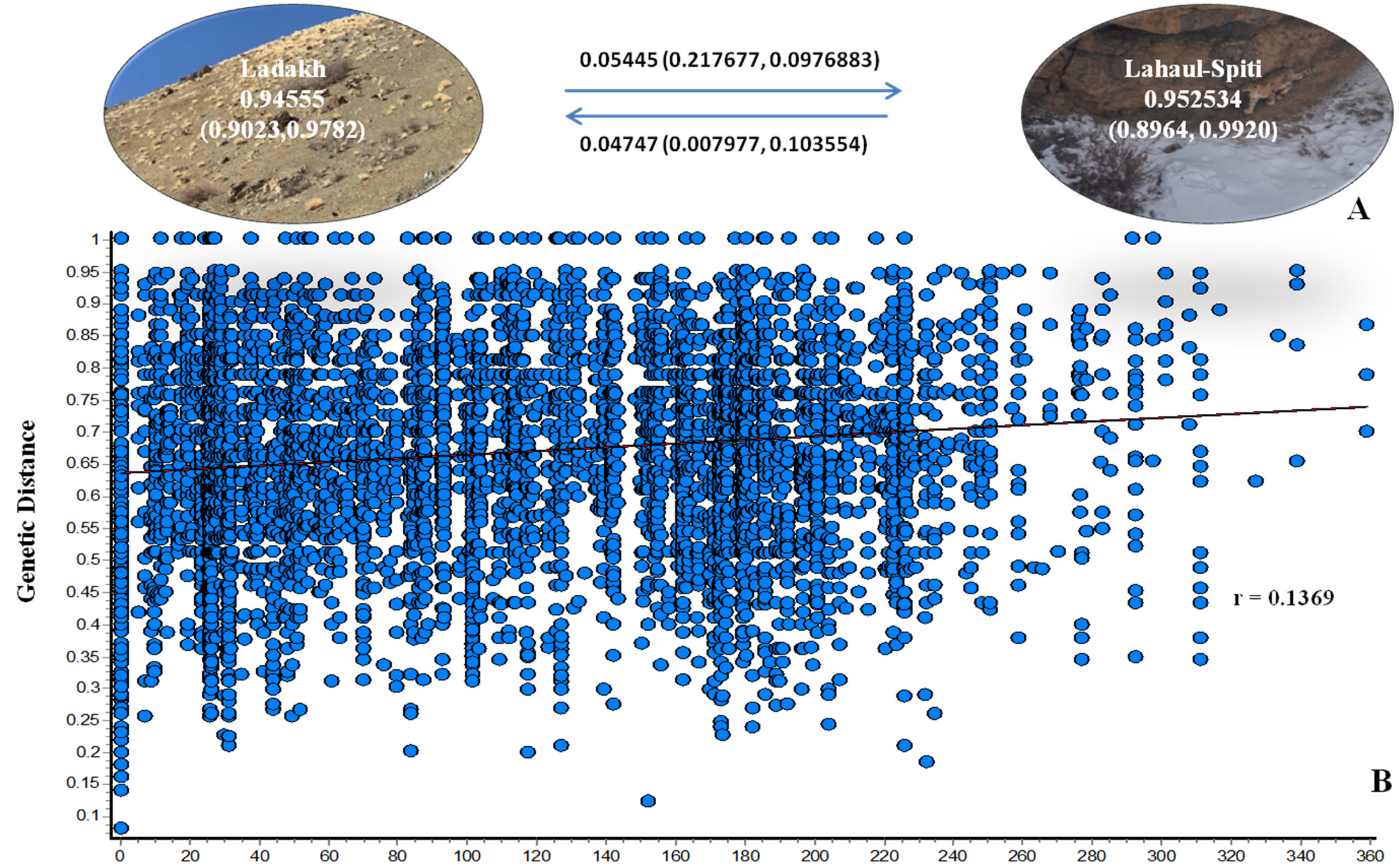

Figure S5 A) Migration rate between the Lahaul-Spiti and Ladakh region by BAYESASS with 0.05 prior of migration rate. B) Mantel test shows Isolation by distance and display not significant relationship between the geographical and genetic distance.

Table S1: Characteristics and reference of microsatellite markers used in standardisation of genotypes in Ibex.

S.No.	Locus	Repeat Motif	Sequence(F) (5'-3')	Sequence(R) (5'-3')	Allele Size Range (bp)	References
1	Haut14	GT	CCAGGGAAGATGAAGTGACC	TGACCTTCACTCATGTTATTAA	120-157	Thieven et al., 1995
2	INRA35	GT	TTGTGCTTTATGACACTATCCG	ATCCTTTGCAGCCTCCACATTC	112-120	Vaiman et al., 1994
3	CSPS115	Inte GT	AAAGTGACACAACAGCTTCTCCAG	AACGAGTGTCCTAGTTTGGCTGTG	238-256	Steffen et al., 1993
4	CSSM19	Inte GT	TTGTCAGCAACTTCTTGTATCTTT	TGTTTTAAGCCACCCAATTATTTG	150-160	Moore et al., (1992,)
5	BM1818	TG	AGTGCTTTCAAGGTCCATGC	AGCTGGGAATATAACCAAAGG	249-272	Bishop et al., 1994
6	CSSM14	GT	AAATGACCTCTCAATGGAAGCTTG	GAATTCTGGCACTTAATAGGATTCA	127-134	Moore et al., (1992,
7	Haut24	GT	CTCTCTGCCTTTTTCCCTGT	AATACACTTTAGGAGAAAAATA	109-126	Thieven et al., 1997
8	BM2113	(TG)	CGT GCC TTC TAC CAA ATA CCC	CTT CCT GAC AGA AGC AAC ACC	133-152	Georges & Massey, 1992
9	TGLA53	$(TG)_6CG(TG_4)(TA)_n$	GCT TTC AGA AAT AGT TTG CAT TCA	ATC TTC ACA TGA TAT TAC AGC AGA	205-220	Solinas-Toldo et al., 1993
10	ETH10	AC	GTT CAG GAC TGG CCC TGC TAA CA	CCT CCA GCC CAC TTT CTC TTC TC	188-215	Vaiman et al., 1994
11	TGLA126	TG	CTA ATT TAG AAT GAG AGA GGC TTC T	TTG GTC TCT ATT CTC TGA ATA TTC C	127-145	Steffen et al., 1993
12	TGLA122	ACnATn	AAT CAC ATG GCA AAT AAG TAC ATA C	AAT CAC ATG GCA AAT AAG TAC ATA C	160-171	Bishop et al., 1994
13	INRA23	AC	GAG TAG AGC TAC AAG ATA AAC TTC	TAA CTA CAG GGTGTT AGA TGA ACT C	239-245	Moore & Byrne 1993
14	ETH3	$(GT)_nCA(GT)_6$	GAACCTGCCTCTCCTGCATTGG	ACT CTG CCT GTG GCC AAG TAG G	148-162	Maudet et., 2002
15	ETH225	$(GT)_4CG(TG)(CA)_n$	GATCACCTTGCCACTATTTCCT	ACA TGA CAG CCA GCT GCT ACT	188-197	Steffen et al., 1993
16	BM1824	GT	GAG CAA GGT GTT TTT CCA ATC	CAT TCT CCA ACT GCT TCC TTG	218-231	Bishop et al.,1994
17	SPS115	$(CA)_nTA(CA)_6$	AAAGTGACACAACAGCTTCTCCAG	AACGAGTGTCCTAGTTTGGCTGTG	145-165	Bishop et al., 1994
18	HEL1	GT)	CAACAGCTATTTAACAAGGA	AGGCTACAGTCCATGGGATT	116-146	Bishop et al., 1994
19	SR-CSRP6	CA	CATAGTTCATTCACAATATGGCA	GTTTCTTCATGGAGTCACAAAGAGTTGAA	134-193	Georges & Massey ,1992
20	ETH152	TA	TACTCGTAGGGCAGGCTGCCTG	GAGACCTCAGGGTTGGTGATCAG	104-131	Georges & Massey, 1992
21	BM203	GTAC	GGGTGTGACATTTTGTTCCC	CTGCTCGCCACTAGTCCTTC	90-135	Solinas-Toldo et al., 1993
22	BM415	GT	GCTACAGCCCTTCTGGTTTG	GAGCTAATCACCAACAGCAAG	115-127	Kaukinen & Varvio 1993

Table S2: Sixteen microsatellite marker panels used for multilocus genotyping of Ibex samples

Marker	dye	size	temp	Multipex
**Haut14**	F	148-159	55	1
**INRA35**	F	90-111		
**CSPS115**	F	212-247		
**INRA23**	V	200-202		
**BM1818**	N	249-259		
**CSSM14**	V	122-128		
**BM1824**	N	163-171		
**TGLA53**	F	150-157	55	2
**ETH152**	F	193-209		
**BM203**	V	195-203		
**SPS115**	N	238-245		
**SR-CSRP6**	F	149-154	57	3
**ETH10**	F	205-206		
**BM415**	F	111-123		
**CSSM19**	F	156	53	4
**ETH225**	V	80-150		

Table S3: Total variables included in Spatial modelling of Habitat suitability of Himalayan Ibex and the variable used after correlation test are indicated by *.

**ENVIRONMENTAL VARIABLES**	
Elevation*	Elevation ≥ 7000 m
Aspect*	Aspect (Direction)
Slope*	Slope (Steepness)
Ruggedness*	Rugged
dst_ road*	Euclidean distance to road
dst_water*	Euclidean distance to water
Hii*	Human Interference Index
Twi*	Topographic Wetness Index

**Land Use Land Cover**	
Grass*	Euclidean distance to grassland
Eusn*	Euclidean distance to Snow cover
Eubr*	Euclidean distance to Barren
**BIOCLIMATIC VARIABLES**	
Bio_1*	Annual Mean Temperature
Bio_2*	Mean Diurnal Range (Mean of monthly (max temp - min temp))
Bio_3*	Isothermality (BIO2/BIO7) (×100)
Bio_4	Temperature Seasonality (standard deviation ×100)
Bio_5	Max Temperature of Warmest Month
Bio_6	Min Temperature of Coldest Month
Bio_7*	Temperature Annual Range (BIO5-BIO6)
Bio_8	Mean Temperature of Wettest Quarter
Bio_9	Mean Temperature of Driest Quarter
Bio_10	Mean Temperature of Warmest Quarter
Bio_11	Mean Temperature of Coldest Quarter
Bio_12	Annual Precipitation
Bio_13	Precipitation of Wettest Month
Bio_14	Precipitation of Driest Month
Bio_15*	Precipitation Seasonality (Coefficient of Variation)
Bio_16	Precipitation of Wettest Quarter
Bio_17*	Precipitation of Driest Quarter
Bio_18	Precipitation of Warmest Quarter
Bio_19	Precipitation of Coldest Quarter

Table S4: Polymorphic sites of each haplotype using the d-loop gene

	13	25	31	53	57	58	60	63	69	73	87	93	113	114	121	124	134	135	146	153	159	160	163	164	165	170	189	190	197	210	226	234	236		
Hap1	A	G	C	A	A	G	A	G	C	A	T	G	G	A	T	T	A	T	A	G	A	G	C	G	A	A	C	A	C	T	T	A	G	C	Lahaul/Kagil(Z)
Hap2	.	A	T	.	.	.	.	.	.	.	.	.	.	.	.	C	.	.	.	.	.	.	.	.	.	.	.	.	.	.	.	.	.	.	Lahaul, Spiti, Leh
Hap3	.	.	.	.	.	.	.	.	.	.	.	.	.	.	.	.	.	.	.	.	.	.	.	.	.	.	.	.	.	.	.	.	C	.	Lahaul
Hap4	.	A	T	.	.	.	.	.	.	G	.	.	.	.	.	C	.	.	.	.	.	.	.	.	.	.	.	.	.	.	.	.	.	.	Lahaul
Hap5	.	.	T	.	.	A	G	.	.	.	.	.	.	G	.	C	.	.	.	.	.	.	.	.	.	.	.	G	.	.	.	.	.	.	Lahaul
Hap6	.	.	T	.	.	.	.	.	.	.	.	.	.	.	.	C	.	.	.	.	.	.	.	.	.	.	.	.	.	.	.	.	.	.	Lahaul, Leh
Hap7	C	.	T	.	.	.	.	A	T	.	.	A	A	G	C	.	.	C	.	.	.	.	T	.	.	G	T	G	.	C	.	G	.	.	Leh
Hap8	C	.	T	G	G	.	.	A	T	.	.	A	A	G	C	.	.	C	.	A	G	.	T	.	G	G	T	.	.	C	.	G	.	.	Leh
Hap9	.	.	T	.	.	.	.	.	.	.	C	.	.	G	.	C	.	.	.	A	.	.	.	.	.	.	.	.	.	C	.	.	.	.	Leh
Hap10	.	.	.	.	.	.	.	.	.	.	.	.	.	.	.	.	.	.	.	.	.	.	.	.	.	.	.	.	.	C	.	.	.	.	Kargil(Z)
Hap11	.	A	.	.	.	.	.	.	.	.	.	.	.	.	.	.	.	.	.	.	.	.	.	.	.	.	.	.	.	.	.	.	.	.	Kargil(Z)
Hap12	.	.	.	.	.	.	.	.	.	.	C	.	.	.	.	C	G	.	.	.	.	.	.	.	.	.	.	.	.	C	.	.	.	G	Kargil(D)
Hap13	.	A	T	.	.	.	.	A	T	.	.	.	.	G	.	.	.	.	.	.	.	.	.	A	.	G	.	.	.	.	.	.	.	.	Leh(N)
Hap14	.	A	T	.	.	.	G	.	.	.	.	.	.	G	.	.	.	.	G	.	.	A	.	.	.	.	.	.	.	C	C	.	.	.	Leh(T)
Hap15	.	A	.	.	.	.	.	.	.	.	.	.	.	.	.	.	.	.	.	.	.	.	.	.	.	.	.	G	.	.	.	.	.	.	Lahaul
Hap16	.	A	T	.	.	A	.	A	.	.	.	.	.	G	.	C	.	.	.	.	.	.	.	.	.	.	.	G	T	.	.	.	.	.	Lahaul

Table S5: Haplotype diversity indices and Neutrality test of Ibex using Cytb and d-loop gene.

Gene	K	H	Hd	S	π	Fu&Li D	Fu&Li F	Tajima
CB (355bp)	0.600	4	0.522±0.050	3	0.00169 ±0.0002	-0.5304 NS, P>0.10	-0.4358 NS, P>0.10	-0.0229 NS, P>0.10
D-loop (330bp)	6.977	16	0.907 ±0.019	34	0.0206 ±0.0025	-0.142 NS, P>0.10	-0.209 NS, P>0.10	-0.233 NS, P>0.10
**K**: average number of nucleotide difference, **H**: No. of Haplotypes, **Hd**: Haplotype diversity, **S:** Polymorphic sites, **π**: Nucleotide diversity								

Table S6: Population genetics indices using ten microsatellite marker in two inferred populations LA-Ladakh and LS-Lahaul-Spiti

Pop	Locus-10	N	Na	Ne	Ho	He	uHe	F
Pop1-LA	Haut14	56	12.000	3.762	0.321	0.734	0.741	0.562
	ETH152	52	13.000	4.630	0.558	0.784	0.792	0.289
	BM415	59	10.000	5.512	0.627	0.819	0.826	0.234
	CSSM14	51	11.000	5.026	0.588	0.801	0.809	0.266
	ETH225	49	10.000	5.214	0.449	0.808	0.817	0.444
	INRA35	44	8.000	3.520	0.500	0.716	0.724	0.302
	BM1824	58	8.000	3.788	0.172	0.736	0.742	0.766
	CSRP6	57	11.000	4.451	0.439	0.775	0.782	0.434
	CSSM19	51	11.000	3.825	0.627	0.739	0.746	0.150
	ETH10	60	10.000	2.351	0.250	0.575	0.580	0.565
LA	Mean	53.700	10.400	4.208	0.453	0.749	0.756	0.401
	SE	1.620	0.499	0.299	0.051	0.022	0.023	0.060
Pop2-LS	Haut14	46	11.000	6.904	0.348	0.855	0.865	0.593
	ETH152	45	12.000	5.146	0.467	0.806	0.815	0.421
	BM415	51	5.000	2.555	0.333	0.609	0.615	0.452
	CSSM14	51	7.000	3.320	0.902	0.699	0.706	-0.291
	ETH225	41	6.000	2.553	0.415	0.608	0.616	0.318
	INRA35	45	8.000	2.705	0.489	0.630	0.637	0.224
	BM1824	46	7.000	2.718	0.348	0.632	0.639	0.450
	CSRP6	49	7.000	2.236	0.367	0.553	0.558	0.335
	CSSM19	45	10.000	2.150	0.178	0.535	0.541	0.668
	ETH10	45	5.000	1.387	0.044	0.279	0.282	0.841
LS	Mean	46.400	7.800	3.167	0.389	0.621	0.627	0.401
	SE	0.980	0.772	0.518	0.071	0.050	0.050	0.096
Total	Mean	50.050	9.100	3.688	0.421	0.685	0.692	0.401
	SE	1.245	0.538	0.315	0.043	0.030	0.031	0.055

N: No. of Alleles; **Na:** No. of observed alleles; Ne: No of effective alleles; Ho: Observed Heterozygosity; He: Expected heterozygosity; uHe: unbiased expected heterozygosity; F: fixation index.

Table S7: First generation migrant individuals which were inferred in different cluster and assigned to another cluster based on Log(L_home/L_max) probability in GeneClass.

S.No	Individual	Home	Assigned (%)	Log(L_home/L_max)
1	MT-2414	LA	LS-84.3	0.73
2	MT-2415	LA	LS-98.23	1.744
3	MT-2416	LA	LS-99.96	3.446
4	MT-2417	LA	LS-99.90	3.041
5	MT-2418	LA	LS-99.98	3.88
6	MT-2465	LA	LS-87.21	0.834
7	MT-2466	LA	LS-70.19	0.372
8	MT-2471	LA	LS-95.86	1.365
9	MT-3410	LA	LS-89.98	0.933
10	MT-3581	LA	LS-68.65	0.34
11	MT-5081	LA	LS-57.96	0.14
12	MT-5105	LA	LS-58.68	0.152
13	MT-5106	LA	LS-57.38	0.129
14	MT-5109	LA	LS-94.70	1.252
15	MT-5111	LA	LS-93.91	1.189
16	MT-5206	LA	LS-99.60	2.404
17	MT-5314	LA	LS-72.39	0.419
18	MT-1565	LS	LA-98.13	1.712
19	MT-1574	LS	LA-53.1	0.054

20	MT-1599	LS	LA-99.90	3.015
21	MT-2943	LS	LA-81.98	0.658
22	MT-2945	LS	LA-76.37	0.51
23	MT-2962	LS	LA-98.39	1.786
24	MT-2990	LS	LA-50.18	0.003
25	MT-ZS393	LS	LA-99.96	3.394
